# Dielectric Tailoring of Perovskite-Polymer Composites for High-Performance Triboelectric Nanogenerators

**DOI:** 10.3390/polym17070969

**Published:** 2025-04-02

**Authors:** Venkatraju Jella, Swathi Ippili, Soon-Gil Yoon

**Affiliations:** Department of Materials Science and Engineering, Chungnam National University, Daedeok Science Town, Daejeon 34134, Republic of Korea; venkatrajujella@gmail.com

**Keywords:** MASnCl_3_, MASnCl_3_–PMMA composite, dielectric, TENG, sensor, power source

## Abstract

The rapid advancement of wearable electronics and the Internet of Things (IoT) has driven the demand for sustainable power sources to replace conventional batteries. In this study, we developed a high-performance, lead-free triboelectric nanogenerator (TENG) using methylammonium tin chloride (MASnCl_3_) perovskite–poly(methyl methacrylate) (PMMA) composite films. MASnCl_3_ was synthesized via an anti-solvent-assisted collision technique and incorporated into a flexible PMMA matrix to enhance dielectric properties, thereby improving triboelectric output. The optimized 10 wt% MASnCl_3_–PMMA composite-based TENG exhibited a maximum output voltage of 525 V, a current of 13.6 µA, and of power of 2.5 mW, significantly outperforming the many halide perovskite-based TENGs. The device demonstrated excellent pressure sensitivity, achieving 7.72 V/kPa in voltage detection mode and 0.2 μA/kPa in current detection mode. The device demonstrated excellent mechanical stability and was successfully used to power a small electronic device. The findings highlight the potential of halide perovskite–polymer composites in developing eco-friendly, efficient mechanical energy harvesters for next-generation self-powered electronics and sensor applications.

## 1. Introduction

The rapid advancement of wearable electronics and the Internet of Things (IoT) has significantly increased the demand for sustainable power sources to overcome the limitations of conventional energy storage devices, such as batteries, which often require frequent recharging and pose environmental challenges upon disposal [1,2,3]. Energy harvesting technologies, which convert ambient energies—including light, mechanical energy, and thermal gradients—into usable electrical power, have gained considerable attention as promising solutions for powering self-sustained and low-power electronic devices [4,5,6]. Among various energy harvesting approaches, mechanical energy harvesters have shown particular promise, with piezoelectric nanogenerators (PENGs) and triboelectric nanogenerators (TENGs) being extensively investigated for their capability to convert mechanical energy into electrical energy [7,8]. TENGs, in particular, have attracted widespread interest due to their versatile energy harvesting potential from diverse mechanical sources such as human motion, wind, and environmental vibrations. This growing interest can be attributed to the advantages of TENGs, including their facile fabrication processes, high power density, and cost-effectiveness [9]. Furthermore, TENGs have demonstrated significant potential as self-powered sensors and sustainable power sources for driving low-power electronics, contributing to the development of autonomous and maintenance-free devices [10,11]. Despite these advantages, the output performance of TENGs remains insufficient for practical applications, necessitating further enhancements in their energy generation capabilities. To address this challenge, numerous studies have focused on fabricating nanocomposites by incorporating functional fillers such as barium titanate (BaTiO_3_), zinc oxide (ZnO), and lead zirconate titanate (PZT) into the triboelectric layers to improve dielectric properties and enhance charge generation [12,13,14]. However, these inorganic fillers often involve complex synthesis procedures, high-temperature processing, and increased production costs, which hinder the scalable and cost-effective development of high-performance TENGs [15,16]. Therefore, there is a growing need to develop alternative materials with excellent dielectric properties through simple and cost-effective approaches. Such materials could offer a viable path toward constructing efficient TENGs, facilitating broader adoption in real-world applications where sustainable and high-output power sources are required.

Recently, halide perovskite materials have garnered significant attention for a wide range of optoelectronic applications, including solar cells, photodetectors, light-emitting diodes (LEDs), and lasers, owing to their simple low-temperature synthesis, excellent optical properties, and tunable bandgap [17,18,19]. Beyond optoelectronics, halide perovskites have also emerged as promising candidates for mechanical energy harvesting applications due to their favorable dielectric and piezoelectric/ferroelectric properties [16,20]. A variety of triboelectric nanogenerators (TENGs) have been developed using both organic and inorganic halide perovskites, demonstrating their potential to convert mechanical energy into electrical energy [21,22,23]. However, the majority of these TENGs utilize lead-based halide perovskite materials, which raises concerns about toxicity and environmental impact, thereby limiting their suitability for wearable and portable device applications [24,25]. Moreover, most studies have focused on fabricating TENGs using only halide perovskites, leading to stability challenges under ambient conditions and high mechanical forces or pressures. These stability issues pose significant barriers to the practical deployment of TENGs, particularly in dynamic and real-world environments. Although tin (Sn)-based perovskite materials exhibit high polarization and excellent dielectric properties, they have been underexplored for TENG applications due to their inherent instability under ambient conditions [26,27]. To address this challenge, developing air-stable Sn-based perovskites and incorporating them into a polymer matrix could provide a viable strategy for fabricating high-performance and stable TENGs. Such advancements could enhance the viability of TENGs for biomechanical energy harvesting and wearable sensor applications, contributing to the broader adoption of sustainable and eco-friendly energy solutions.

In this work, eco-friendly and lead-free methylammonium tin chloride (CH_3_NH_3_SnCl_3_, MASnCl_3_) synthesized via an anti-solvent-assisted collision technique was utilized to develop a high-performance triboelectric nanogenerator (TENG) by incorporating it into a flexible poly(methyl methacrylate) (PMMA) polymer matrix. The surface morphology, crystallinity, and dielectric behavior of the composite films were systematically analyzed. Among various loadings, the 10 wt% MASnCl_3_-PMMA composite exhibited significantly enhanced triboelectric output due to its superior dielectric properties. Furthermore, the developed TENG was demonstrated as a self-powered pressure sensor and an energy harvester capable of charging capacitors to power small-scale electronic devices.

## 2. Materials and Methods

### 2.1. Materials

Tin(II) chloride (SnCl_2_, anhydrous, 99%, Alfa Aesar, Haverhill, MA, USA), and methylammonium chloride (MACl, 98%, Sigma Aldrich, St. Louis, MO, USA); Toluene (anhydrous, 99.8%, Alfa Aesar); Diethyl ether (≥99.0%, anhydrous, ACS reagent, contains BHT as an inhibitor, Sigma Aldrich); PMMA (Poly(methyl methacrylate), Sigma Aldrich); PTFE target (iTASCO, Seoul, Republic of Korea); indium tin oxide (ITO)-coated polyethylene terephthalate (PET) substrates (iTASCO, Republic of Korea). All the purchased chemicals were used without any further purification.

### 2.2. Synthesis of MASnCl_3_ Perovskite Powder

MASnCl_3_ (CH_3_NH_3_SnCl_3_) perovskite was synthesized using the anti-solvent-assisted collision technique (ACT) as described in our previous studies [27,28]. Specifically, an equimolar (0.1 M) ratio of SnCl_2_ and MACl was added to a vial containing 3 mL of diethyl ether, an anti-solvent with low polarity. The mixture was vigorously stirred at 50 °C for 24 h on a hot plate under ambient air conditions. After the reaction, the resulting solution was filtered, and the white solid product was dried in an oven to obtain the MASnCl_3_ perovskite powder. To enhance the stability of perovskite, the powder was further annealed at 180 °C for 30 min using a rapid thermal annealing system under an argon atmosphere.

### 2.3. Fabrication of MASnCl_3_–PMMA Polymer Composites and TENGs

To fabricate MASnCl_3_–PMMA polymer composites, the annealed MASnCl_3_ powder was incorporated into a polymethyl methacrylate (PMMA) polymer solution at various loadings (5, 7, 10, 12, and 15 wt%). Initially, the PMMA solution was prepared by dissolving 150 mg of PMMA in toluene under continuous stirring at 60 °C overnight to ensure complete dissolution. Subsequently, the desired amounts of MASnCl_3_ powder were added to 1 mL of the PMMA solution, and the mixture was further stirred at 60 °C for an additional 2 h on a hot plate to achieve a homogeneous composite solution. The resulting composite solution was then spin-coated onto cleaned substrates, including glass, ITO-PET, and FTO-coated glass, to prepare thin films for further analysis. The coated films were dried at 60 °C for 5 h on a hot plate to remove residual solvent and enhance film stability.

To fabricate the TENGs, the composite films spin-coated onto ITO-coated PET substrates were utilized. Copper wires were connected to the electrodes using carbon tape to ensure effective electrical contact. For the counter electrode, a polytetrafluoroethylene (PTFE)-coated PET/ITO substrate was employed. In this work, the PTFE was deposited using an on-axis radio frequency (RF) sputtering technique with a PTFE target. The sputtering process was conducted at a power of 80 W and a working pressure of 5 mTorr. Finally, the PTFE-coated electrode was assembled with the MASnCl_3_–PMMA/ITO/PET samples by positioning the PTFE layer to face the MASnCl_3_–PMMA composite, maintaining a 5 mm gap between the layers. This configuration was used to construct the complete TENG device, facilitating subsequent performance evaluation and testing. The size and active area of the TENGs are 3 × 3 cm^2^ and 1 × 1 cm^2^, respectively.

### 2.4. Characterization

The crystallinity and phase of the fabricated perovskite and composite films were analyzed using D2 Phaser (Bruker, Billerica, MA, USA) XRD equipped with Cu radiation (30 kV, 10 mA) and a Ni filter. The morphology and thickness, and elemental distributions of desired samples were examined by field emission scanning electron microscopy (FE-SEM, TESCAN CLARA). The dielectric properties (capacitance and dissipation factor) of the composite films were studied using an impedance/gain-phase analyzer (HP4194A) with a metal-insulator-metal (MIM) structure with a 150 μm diameter DC-sputtered Pt (100 nm) top electrode and an FTO-glass bottom electrode. The output performance of TENGs was measured under mechanical force using a custom-made mechanical pushing tester. TENGs were tested in continuous vertical contact-separation mode. The open-circuit voltage and short-circuit current were recorded using a digital phosphor oscilloscope (DPO 5204B, Tektronix, Beaverton, OR, USA) and a low-noise current amplifier (SR570, Stanford Research Systems, Sunnyvale, CA, USA), respectively, with a probe impedance of 40 MΩ. The applied force was monitored by a load cell (Dacel UU-K20).

## 3. Results and Discussion

**Figure 1:**
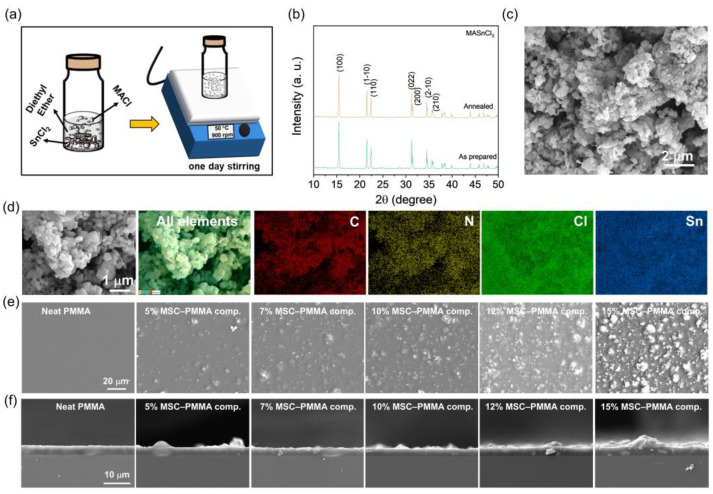
(**a**) Schematic illustration of the synthesis of MASnCl_3_ perovskite powder using the ACT method, and (**b**) XRD pattern of as-prepared and 180 °C–annealed MASnCl_3_ powders. (**c**) SEM morphology, (**d**) SEM–EDS analysis of annealed MASnCl_3_ powder, (**e**) SEM morphologies, and (**f**) thicknesses of MASnCl_3_–Poly(methyl methacrylate) (MSC–PMMA) composite films with various loading amounts of MASnCl_3_ perovskite in the PMMA matrix.

An eco-friendly, lead-free MASnCl_3_ (MSC) perovskite was synthesized using an anti-solvent-assisted collision technique, as schematically illustrated in Figure 1a. The X-ray diffraction (XRD) pattern of both the as-prepared and annealed MSC powders at 180 °C is presented in Figure 1b. The annealing process serves to eliminate unwanted phases in the perovskite material and enhance its structural stability [27,28]. The dominant crystallographic planes observed in the XRD patterns correspond to the distorted triclinic system with a P1 space group, confirming the crystalline nature of MASnCl_3_ perovskite [29,30,31]. The surface morphology of the annealed MSC perovskite powder was analyzed using scanning electron microscopy (SEM), revealing a nanoparticle-like structure (Figure 1c). Additionally, SEM-energy dispersive spectroscopy (EDS) images demonstrate the uniform distribution of all expected elements, such as carbon (C), nitrogen (N), chlorine (Cl), and tin (Sn), further validating the material’s composition (Figure 1d).

**Figure 2:**
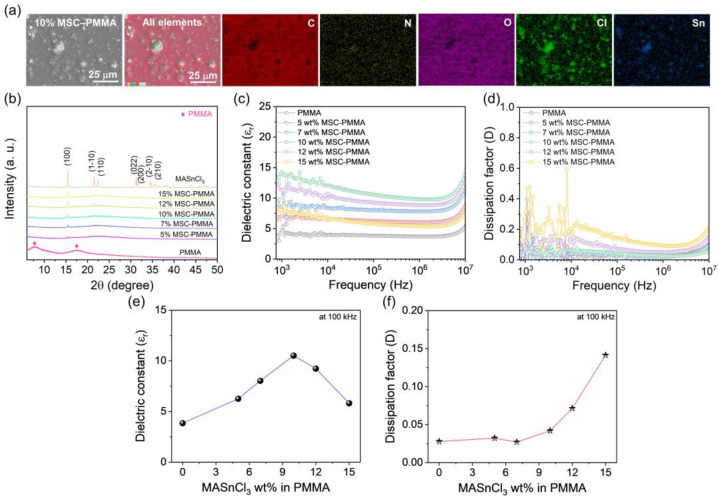
(**a**) SEM–EDS analysis of annealed 10 wt% MSC–PMMA composite film, (**b**) XRD pattern of MSC–PMMA composite films with various loading amounts of MASnCl_3_ perovskite in the PMMA matrix. Frequency-dependent (**c**) dielectric constant and (**d**) dissipation factor of MSC–PMMA composite films with different MASnCl_3_ loading amounts. (**e**,**f**) Summary of the dielectric constant and dissipation factor values obtained at 100 kHz frequency as a function of wt%.

Various amounts of MSC powder were mixed with PMMA polymer to fabricate flexible MASnCl_3_–PMMA (MSC–PMMA) composite films using a spin-coating technique. The morphological variations in MSC–PMMA composite films, depending on the MSC loading, are clearly observed in the SEM images shown in Figure 1e. Notably, the neat PMMA polymer film exhibits a smooth surface. However, in MSC–PMMA composite films, MSC particles are uniformly distributed throughout the polymer matrix. Additionally, the gaps between the particles decrease with increasing MSC loading. At higher loading levels (≥12 wt%), the gaps become significantly narrower, and noticeable particle agglomeration is observed. Further, all the spin-coated MSC–PMMA composite films, including the neat PMMA film, exhibited an average thickness of ~3 μm (Figure 1f). Moreover, SEM-EDS analysis of the 10 wt% MSC–PMMA composite film confirms the uniform distribution of MSC perovskite within the polymer matrix. The expected elemental composition, including C, N, O, Cl, and Sn, is clearly observed, further validating the homogeneous dispersion of perovskite in the polymer (Figure 2a). This uniform dispersion plays a crucial role in optimizing the dielectric properties of the composite, which directly influences its energy-harvesting efficiency. In addition, the XRD patterns of the MSC–PMMA composite films are presented in Figure 2b. The neat PMMA film exhibited broad peaks, indicating its amorphous nature [32]. In contrast, the composite films displayed distinct, intense, and sharp peaks corresponding to the crystalline MASnCl_3_ perovskite phase [29,30,31]. Notably, no unwanted phases or residual starting material phases were detected, confirming that the perovskite remains highly stable within the polymer matrix. This stability is crucial for maintaining the structural integrity and performance of the composite films in energy-harvesting applications. Furthermore, the dielectric properties of the MSC–PMMA composite films, along with neat PMMA, were investigated under varying frequencies (Figure 2c,d). The summarized dielectric constant and dissipation factor values of the corresponding films, measured at a frequency of 100 kHz, are presented in Figure 2e,f. These values further illustrate the influence of MSC loading on the dielectric properties of the composite films. The neat PMMA polymer exhibited a dielectric constant of 3.85 with a dissipation factor of 0.027. However, with increasing MASnCl_3_ loading, the dielectric constant gradually increased, reaching a maximum value of 10.5 with a dissipation factor of 0.04 for the 10 wt% composite sample. This enhancement in dielectric properties can be attributed to the Maxwell–Wagner–Sillars (MWS) interfacial polarization effect, where charge carriers accumulate at the interfaces between the high-dielectric MSC perovskite particles and the PMMA polymer matrix, leading to an increase in the overall dielectric constant [21,33,34]. This enhancement is also due to the high polarizability of the MSC perovskite, which facilitates improved charge accumulation and transfer within the composite. However, when the MSC loading exceeded 12 wt%, the dielectric constant began to decrease, while the dissipation factor increased significantly. This decline in dielectric performance at higher loadings is likely due to increased dielectric loss and perovskite agglomeration, which can disrupt the uniform charge distribution and lead to charge dissipation, ultimately reducing the effectiveness of the composite for triboelectric applications [35].

**Figure 3:**
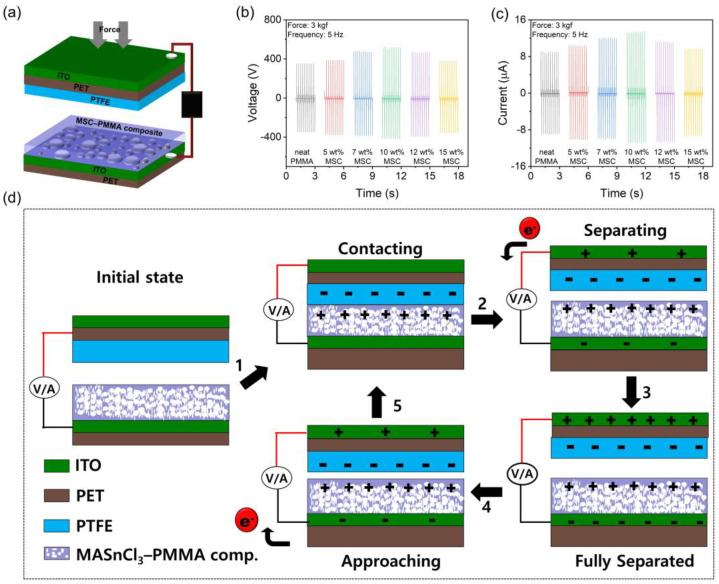
(**a**) Schematic diagram of MSC–PMMA composite-based triboelectric nanogenerator. Triboelectric output (**b**) voltage and (**c**) current signals of the MSC–PMMA composite–based TENGs with varying MSC loading amounts in the PMMA matrix. (**d**) The schematic working mechanism of the MSC–PMMA composite-based TENG.

The mechanical energy harvesting capability of the prepared eco-friendly, lead-free MSC–PMMA composite films was evaluated by fabricating a flexible TENG (Figure 3a) and measuring its electrical output in a vertical contact-separation mode under a mechanical force of 3 kgf at a frequency of 5 Hz. The neat PMMA-based TENG exhibited a triboelectric output voltage of 350 V and a current of 9.1 μA, with the positive signal appearing downward and the release signal appearing upward (Figure 3b,c). This behavior indicates the inherently positive triboelectric nature of the PMMA surface relative to the counter PTFE polymer surface [35,36,37]. However, incorporating MASnCl_3_ perovskite into the PMMA polymer matrix led to a gradual enhancement in triboelectric performance with increasing MASnCl_3_ loading. Notably, the 10 wt% MSC–PMMA composite-based TENG exhibited a significantly higher output voltage of 525 V and a current of 13.6 μA while maintaining the same signal direction as the neat PMMA-based TENG. The enhancement in the triboelectric output observed in the composite films can be attributed to the improved dielectric properties of the MSC–PMMA composites [20,21,38,39,40,41]. A higher dielectric constant enhances the material’s ability to store and sustain charge carriers, leading to an increased surface charge density during the contact-separation process. This, in turn, strengthens the electrostatic induction effect between the triboelectric layers, resulting in greater charge transfer and improved overall triboelectric performance. Furthermore, the incorporation of MASnCl_3_ perovskite into the PMMA matrix likely induces additional polarization effects, further contributing to charge accumulation at the interface [33,34,42,43,44]. These synergistic effects collectively lead to a significant boost in the triboelectric output voltage and current of the TENG devices [21,40,45]. However, with a higher loading amount (≥12 wt%), the triboelectric output declines due to a reduction in dielectric properties and an increase in dielectric loss. The increased dielectric loss leads to charge dissipation rather than effective charge accumulation, thereby reducing the overall output. Additionally, excessive filler content can disrupt the uniformity of the composite, causing agglomeration and defects that hinder charge transfer efficiency [40,46,47].

**Figure 4:**
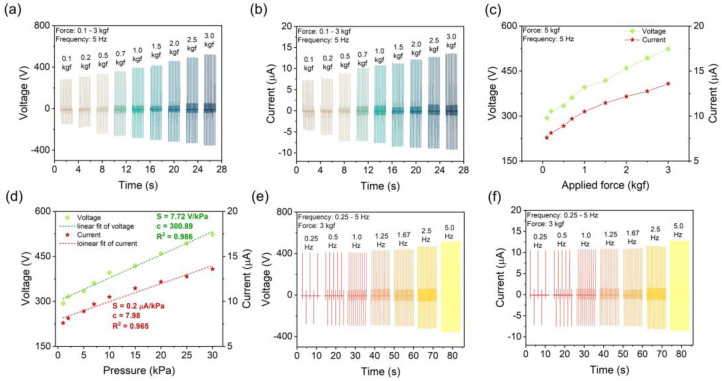
Force–dependent triboelectric output: (**a**) voltage and (**b**) current signals of the 10 wt% MSC–PMMA composite-based TENG. (**c**) The corresponding summarized peak output amplitudes depending on force. (**d**) The pressure–sensitivity plot of the pressure–dependent output plots. Frequency–dependent triboelectric output: (**e**) voltage and (**f**) current signals of the 10 wt% MSC–PMMA composite–based TENG.

The working mechanism of the TENG is schematically illustrated in Figure 3d. The operation of the TENG with MSC–PMMA and PTFE dielectric layers is based on triboelectrification and electrostatic induction. Under applied force, MSC–PMMA film and PTFE come into contact, and electrons transfer from MSC–PMMA to PTFE due to their different electron affinities, with PTFE acquiring negative charges and MSC–PMMA becoming positively charged. Upon separation due to the withdrawal of applied force, an electric potential difference is created, inducing electron flow through an external circuit by generating an electrical signal. When the external force is reapplied, the dielectric layers come back into close proximity. As they approach, the electrostatic potential difference decreases, causing electrons in the external circuit to move back in the opposite direction, resulting in an opposite polarity signal. Incorporating high-dielectric fillers like MASnCl_3_ into PMMA further boosts charge density, leading to improved triboelectric output. In addition, the effect of applied pressure and frequency on the triboelectric output performance of the 10 wt% MSC–PMMA composite-based TENG was investigated. It was observed that the triboelectric output signals gradually increased with increasing applied force from 0.1 kgf to 3 kgf at a constant frequency of 5 Hz (Figure 4a,b). The summarized peak output values are presented in Figure 4c. Linear fitting of the corresponding voltage and current plots resulted in a pressure sensitivity of 7.72 V/kPa in voltage detection mode and 0.2 μA/kPa in current detection mode (Figure 4d). Furthermore, an increase in frequency also led to enhanced triboelectric output performance, with the highest output observed at 5 Hz (Figure 4e,f).

**Figure 5:**
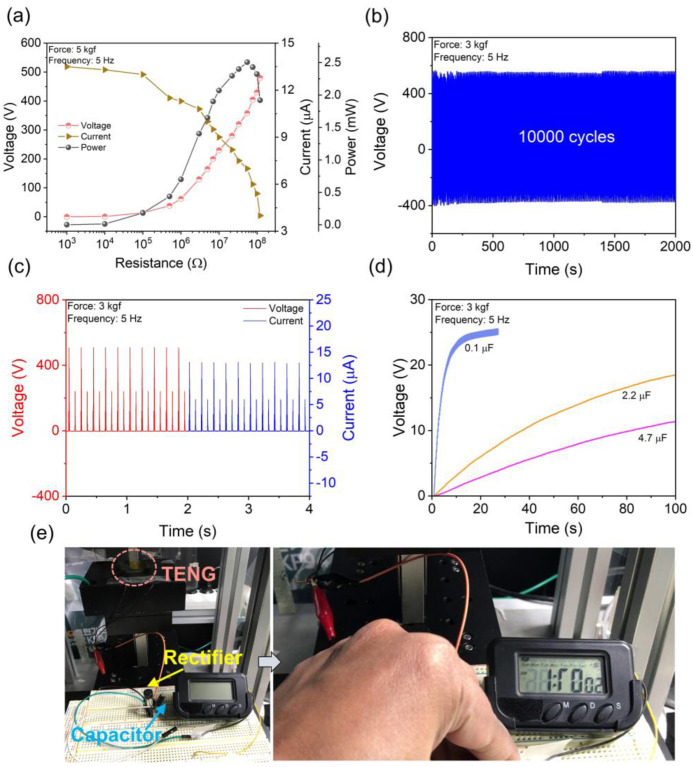
(**a**) Load resistance–dependent triboelectric output voltage, current, and power of the 10 wt% MSC–PMMA composite–based TENG. (**b**) Mechanical durability test of the 10 wt% MSC–PMMA composite–based TENG. (**c**) The rectified triboelectric outputs of the 10 wt% MSC–PMMA composite–based TENG. (**d**) The capacitor charging profiles by the same TENG. (**e**) Operation of a portable watch powered by the 4.7 μF capacitor after charging by the 10 wt% MSC–PMMA composite–based TENG.

To evaluate the practical applicability of the MSC–PMMA composite-based TENG, the maximum output power of the optimized 10 wt% MSC–PMMA composite-based TENG was investigated by connecting various load resistors ranging from 1 kΩ to 0.12 GΩ (Figure 5a). The output voltage and current signals were measured under an applied force of 3 kgf and a frequency of 5 Hz. It was observed that as the load resistance increased, the output voltage gradually increased while the current decreased. The corresponding power output analysis revealed a maximum power of 2.5 mW (0.625 mW/cm^2^) at an optimal matching load resistance of 55 MΩ. The generated triboelectric power is notably higher than many reported halide perovskite/halide perovskite–polymer composite-based nanogenerators (Table 1). In addition, the mechanical robustness of the device was evaluated by continuously measuring its output performance over 10,000 cycles under an applied force of 3 kgf and a frequency of 5 Hz (Figure 5b). The device maintained nearly identical output levels without any noticeable decline, demonstrating the excellent durability and stability of the TENG under prolonged mechanical strain. Moreover, to demonstrate the TENG’s capability as a power source, it was connected to a bridge rectifier to convert AC signals into DC signals, which were then used to store energy in various capacitors. The rectified DC-output signals generated under an applied force of 3 kgf and a frequency of 5 Hz are presented in Figure 5c. The capacitor charging profiles are shown in Figure 5d, where a 0.1 μF capacitor was charged up to 25 V in 20 s, while a 4.7 μF capacitor reached 11.2 V in 100 s using the same TENG. Furthermore, the charged 4.7 μF capacitor was successfully utilized to operate a portable electronic watch, as captured in the snapshot taken during operation (Figure 5e). The findings highlight the potential of this eco-friendly MASnCl_3_ material in advancing efficient mechanical energy harvesters, paving the way for next-generation self-powered electronic systems.

## 4. Conclusions

An eco-friendly, lead-free MASnCl_3_–PMMA composite-based TENG was successfully fabricated and demonstrated for high-performance mechanical energy harvesting. The incorporation of MASnCl_3_ into the PMMA matrix enhanced the dielectric properties, leading to significantly improved triboelectric output. The optimized composite with 10 wt% MASnCl_3_ exhibited a maximum output voltage of 525 V and a current of 13.6 μA, outperforming the neat PMMA-based TENG. The power output characteristics indicated a peak power of 2.5 mW at a matched load resistance of 55 MΩ, confirming its potential for practical applications. Furthermore, the device demonstrated excellent pressure sensitivity, with a voltage detection sensitivity of 7.72 V/kPa and a current detection sensitivity of 0.2 μA/kPa. Additionally, its ability to charge capacitors efficiently and power small electronic devices, such as a portable electronic watch, highlights its feasibility as a sustainable energy source for self-powered electronics and wearable applications.

## Figures and Tables

**Table 1:** Comparison of triboelectric output performance of MASnCl_3_–PMMA composite–based TENG with reported halide perovskite//halide perovskite–polymer composite–based TENGs.

Perovskite	Pressure/ Frequency	Voltage (V)	Current/Current Density	Power/Power Density (mW/cm^2^)	Ref.
Cs_3_Bi_2_Br_9_–PVDF-HFP–SEBS NFs	30 N/5 Hz	400	1.63	0.23 mW/cm^2^	[24]
CS_0_._05_FA_0_._7_MA_0_._25_PbI_3_	50 N/3 Hz	200	16.3 µA	1.13 mW/cm^2^	[48]
MAPbI_3_-PVDF	300 kPa/5 Hz	44.7	4.34 µA/cm^2^	0.059 mW/cm^2^	[42]
CsPbBr_3_–Au	-/2 Hz	240	4.13 µA/cm^2^	0.331 mW/cm^2^	[49]
CsPbBr_3_–Pt (0.5%)	-/-	273	30.3 µA/cm^2^	0.129 mW/cm^2^	[50]
CsPbBr_2.6_I_0.4_	-/0.5 Hz	192	16.7 µA	0.12 mW/cm^2^	[51]
CsPbIBr_2_	-/0.5 Hz	243	3.1 µA/cm^2^	0.204 mW/cm^2^	[52]
CsPb_0.91_Ba_0.09_Br_3_	-/-	220	2.28 µA/cm^2^	0.307 mW/cm^2^	[46]
CsPbCl_3_	10 N/-	257	27.87µA/cm^2^	0.304 mW/cm^2^	[53]
DAPPbI_4_–PVDF	30 kPa/5 Hz	662	18.7 µA/cm^2^	4.28 mW/cm^2^	[21]
CsBi_3_I_10_	50 N/1 Hz	158	4.55 µA/cm^2^	0.38 mW/cm^2^	[54]
MA_2_Bi_2_I_9_–SEBS	20 kPa/5 Hz	537	13.2 µA/cm^2^	3.04 mW/cm^2^	[41]
MASnCl_3_–PMMA (10 wt%)	3 kgf/5 Hz	525	13.6 µA	2.5 mW or (0.625 mW/cm^2^)	This work

## Data Availability

The original contributions presented in this study are included in the article. Further inquiries can be directed to the corresponding authors.

## Abbreviations

IoT	Internet of things
PENG	Piezoelectric nanogenerator
TENG	Triboelectric nanogenerator
PZT	Lead zironate titanate
LEDs	Light emitting diodes
PMMA	Poly (methyl methacrylate)
MACl	Methyl ammonium chloride
PTFE	Polytetra fluoroethylene
ITO	Indium tin oxide
PET	Polyethylene terephthalate
ACT	Anti-solvent-assisted collision technique
FTO	Florine doped tin oxide
MIM	Metal insulator metal
MSC	Methyl ammonium stannous chloride
XRD	X-ray diffraction
SEM	Scanning electron microscopy
MWS	Maxwell–Wagner–Sillars
